# Sexually Transmitted Infection Prevalence and Determinants Among Sexual Health Clinic Attendees in Bucharest, Romania

**DOI:** 10.1111/ijd.70344

**Published:** 2026-02-15

**Authors:** Andrei Tanasov, George‐Sorin Tiplica

**Affiliations:** ^1^ Carol Davila University of Medicine and Pharmacy Bucharest Romania; ^2^ 2nd Department of Dermatology Colentina Clinical Hospital Bucharest Romania

**Keywords:** knowledge‐attitudes‐practices, sexually transmitted infections, MSM sexual health, Romania sexual health, risk perception, *Neisseria gonorrhoeae*, syphilis, screening campaign, STI prevalence, STI surveillance

## Abstract

**Background:**

Sexually transmitted infections (STIs) remain a significant public health concern in Romania, yet limited data exist on STI prevalence and knowledge, attitudes, and practices (KAP) among at risk populations. This study aims to assess STI prevalence and KAP among attendees of a free‐of‐charge STI testing campaign and to examine associations between knowledge, risk behaviors, and laboratory‐confirmed STI status.

**Methods:**

A cross‐sectional study was conducted at Colentina Clinical Hospital, Bucharest, from June to November 2025 during a free‐of‐charge STI testing campaign. Participants (*n* = 70) completed a structured KAP questionnaire using 7‐point Likert scales and underwent comprehensive polymerase chain reaction (PCR) testing from multiple anatomical sites for bacterial STIs (including *Chlamydia trachomatis* , *Neisseria gonorrhoeae* , *Mycoplasma genitalium* , *Ureaplasma urealyticum* ), plus serological testing for syphilis (VDRL/TPHA), human immunodeficiency virus (HIV), and hepatitis B and C.

**Results:**

The sample was predominantly male (84.3%) and men who have sex with men (MSM, 70.0%), with a mean age of 33.3 ± 11.3 years. Despite high overall knowledge (85.3%), STI prevalence was substantial: active syphilis 17.7%, HIV 11.5%, and *Chlamydia trachomatis* 8.6%, while *Ureaplasma urealyticum* was the most commonly‐detected organism (21.4%). Overall, 35.5% had clinically relevant bacterial STIs. Participants with STIs showed significantly higher agreement with specific misconceptions. *Chlamydia trachomatis* was significantly more frequent in non‐MSM (23.8%) than MSM (2.0%, *p* = 0.008), while *Neisseria gonorrhoeae* infections (5.7%) occurred only in MSM, with 80% isolates detected extragenitally. Preference for quarterly screening dropped from 62.9% (if free‐of‐charge) to 17.1% (if paid) (*p* = 0.002).

**Conclusions:**

In this first comprehensive STI study among a predominantly MSM cohort in Romania, prevalence was high (35.5% for clinically relevant bacterial STIs), with multisite testing proving essential. Despite high general knowledge, specific misconceptions predicted positive STI status, while cost remained a major barrier to optimal testing frequency. These findings support implementation of free‐of‐charge, multisite screening programs and targeted interventions addressing specific KAPs.

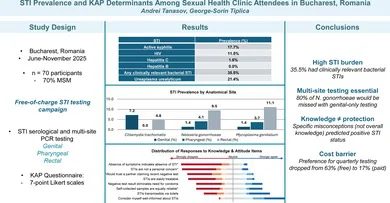

## Graphical abstract

Cross‐sectional study among 70 sexual health clinic attendees (70% MSM) in Bucharest combining multisite STI testing with knowledge, attitudes, and practices assessment. Key findings include 35.5% bacterial STI prevalence, essential extragenital testing, and cost barriers to screening uptake.

## 1 Introduction

Sexually transmitted infections (STIs) represent a significant global public health challenge, with the World Health Organization (WHO) estimating more than 374 million new cases of four curable bacterial STIs annually [1]. Despite effective diagnostic methods and inexpensive treatments, global control targets remain unmet, and many countries have reported substantial increases in notification rates over the past decade [2].

Romania presents a particular epidemiological picture that warrants examination. The 2024 national surveillance report documented only 39 gonorrhea cases (incidence 0.20/100,000), 24 genital *Chlamydia trachomatis* infections (incidence 0.12/100,000), and 574 syphilis cases (incidence 3.01/100,000). These figures contrast sharply with Central and Eastern European nations sharing comparable historical and socioeconomic contexts. Notably, 30 of Romania's 41 counties reported zero gonorrhea cases, and all nationally reported cases were genitourinary (no extragenital infection was reported). Furthermore, only 22 of the 39 gonorrhea cases had documented detection methods, and concurrent human immunodeficiency virus (HIV) and hepatitis testing remained incomplete for most patients [3].

Multiple factors likely contribute to this surveillance gap: limited nucleic acid amplification testing (NAAT) availability outside major urban centers, absence of systematic screening programs for at‐risk populations, reliance on syndromic management without laboratory confirmation, stigma, and inadequate reporting infrastructure [4].

The true burden of STIs in Romania remains to be discovered. Beyond infrastructure limitations, significant underreporting occurs because many physicians do not report diagnosed cases, no mechanisms verify reporting compliance, and private practitioners frequently assume they are exempt from reporting obligations when, in fact, all STI cases should be reported regardless of practice setting. The absence of a dedicated national STI surveillance network compounds these challenges.

Addressing these gaps requires innovative approaches to service delivery. Recent evidence demonstrates that free‐of‐charge, convenient sexual health services can substantially expand access to diagnosis and treatment, particularly for underserved populations and those facing barriers to traditional healthcare settings [5]. Similar initiatives have proven effective elsewhere in Europe, including community‐based centers for MSM, where prevalence studies consistently identify substantial proportions of previously undiagnosed infections [6].

MSM remain disproportionately affected by STIs across Europe, with elevated rates of STIs compared to the general population [7]. Romanian national surveillance data for 2024 revealed only 53 syphilis cases among MSM (9.4% of reported cases) [3], despite MSM comprising a much higher proportion in most Western European countries. This discordance may reflect limited access to STI services among Romanian MSM, influenced by persistent stigma, discrimination, and the absence of dedicated sexual health services for this population. Additionally, in Romania, extragenital testing is not routinely available and remains largely inaccessible even to populations at highest risk. This diagnostic limitation compounds the underreporting problem, as infections at extragenital sites, which may serve as important reservoirs for ongoing transmission, remain invisible to surveillance systems.

KAP studies provide essential insights into STI transmission determinants and serve as a foundation for evidence‐based prevention strategies. Understanding the relationship between knowledge and actual infection status is crucial, as the assumption that increased knowledge automatically translates to protective behaviors oversimplifies the relationship between knowledge and behavior [8].

To address these knowledge and service gaps, we organized a free‐of‐charge STI screening campaign at a sexual health clinic in Bucharest. Our objectives were to: (1) assess STI prevalence including extragenital testing for at‐risk participants; (2) evaluate STI‐related KAP among clinic attendees; (3) examine associations between KAP factors and laboratory‐confirmed STI status; and (4) evaluate attitudes toward self‐collection methods and screening preferences, in order to inform future service delivery models in the Romanian context. This work constitutes a component of the URGENT‐STI research project (PPRC‐EADV‐2024‐0024), a multicenter European initiative investigating antimicrobial resistance in gonorrhea and optimizing STI screening strategies.

## 2 Materials and Methods

### 2.1 Study Design and Setting

This cross‐sectional study was conducted at the 2nd Department of Dermatology and Venereology of Colentina Clinical Hospital, a tertiary care hospital and a reference sexual health center in Bucharest, Romania. As part of a free‐of‐charge STI testing campaign organized to increase screening access, consecutive patients presenting for STI screening or consultation were invited to participate between June and November 2025.

### 2.2 Participants

Inclusion criteria were: age ≥ 18 years, ability to provide informed consent, STI risk factors (clinical suspicion of STIs, unprotected vaginal and/or anal intercourse within the past 3 months, contact with a partner confirmed with STIs, previous diagnosis of STIs, sex work, substance use, sexual minority status, or migrant background) and willingness to complete the questionnaire and undergo extensive STI screening. Exclusion criteria included inability to understand the questionnaire, current antibiotic treatment, or refusal to provide biological samples. A total of 70 participants were enrolled during the study period.

### 2.3 KAP Questionnaire

A structured questionnaire was developed for this study, comprising demographic items, sexual behavior questions, and 33 knowledge and attitude items measured using 7‐point Likert scales (1 = strongly disagree to 7 = strongly agree). The questionnaire was administered in Romanian, and an English translation was provided for two participants.

Items assessed STI‐related knowledge, attitudes toward healthcare and prevention, and common misconceptions. Seventeen items were dichotomized based on responses indicating favorable STI awareness and prevention orientation: for statements reflecting accurate knowledge or protective attitudes, responses of 5–7 (agree) were coded as 1 (favorable); for misconceptions, responses of 1–3 (disagree) were coded as 1 (favorable, indicating rejection of the misconception). This yielded a composite score ranging from 0 to 17, representing the number of favorable responses across knowledge and attitudinal domains (Table S1). The remaining 16 items were retained as continuous Likert variables.

High partner count was defined as ≥ 5 partners in the past 6 months; inconsistent condom use as “never,” “rarely,” or “sometimes”; and early sexual debut as first intercourse before age 18. Preferred screening frequency was assessed separately for free‐of‐charge and paid testing scenarios.

### 2.4 Laboratory Testing

All participants underwent comprehensive STI testing as part of the free‐of‐charge testing campaign. Biological samples were collected from genital, pharyngeal, and rectal sites as appropriate based on reported sexual practices. Genital specimens consisted of first‐void urine for all participants. Extragenital testing (pharyngeal and/or rectal) was offered to participants reporting oral or anal sexual practices. Molecular testing was performed using both multiplex PCR platforms and GeneXpert point of care testing for *Chlamydia trachomatis* and *Neisseria gonorrhoeae* . Complete PCR panels included testing for *Mycoplasma genitalium* , *Mycoplasma hominis* , *Ureaplasma urealyticum* , *Ureaplasma parvum* , and *Trichomonas vaginalis*. Serological testing included syphilis using Venereal disease research laboratory (VDRL) and Treponema pallidum haemagglutination (TPHA) testing, HIV testing using 4th generation antigen/antibody assays, hepatitis B surface antigen, and hepatitis C antibodies. Active syphilis was defined as concurrent TPHA and VDRL positivity (any reactive titre ≥ 1:2) in the absence of documented previous treatment. Participants with isolated TPHA positivity and negative VDRL were classified as past or treated infection. Participants with equivocal VDRL results (+/−) and documented treatment history were also classified as past infection.

Clinically relevant bacterial STI was defined as any positive result for *Chlamydia trachomatis* , *Neisseria gonorrhoeae* , *Mycoplasma genitalium* , or active syphilis.

### 2.5 Statistical Analysis

Statistical analyses were performed using IBM SPSS Statistics version 29. Descriptive statistics were calculated for all variables. Continuous variables were assessed for normality using Shapiro–Wilk tests. Given non‐normal distributions, non‐parametric tests were used throughout. Group comparisons were made using Mann–Whitney U tests for continuous variables and chi‐square tests (or Fisher's exact test when expected cell counts were < 5) for categorical variables. Spearman's rank correlation coefficients were calculated to assess relationships between continuous variables. Statistical significance was set at *p* < 0.05 (two‐tailed). Comparisons of individual KAP items between participants with and without clinically relevant bacterial STI were exploratory. Given the hypothesis‐generating nature of these analyses and the limited sample size, correction for multiple comparisons was not applied; findings are presented with unadjusted *p* values and should be interpreted cautiously.

### 2.6 Ethical Considerations

The study protocol was approved by the Research Ethics Committee of Colentina Clinical Hospital (approval Nr. 2/17.01.2025) and the Scientific Research Ethics Committee of Carol Davila University of Medicine and Pharmacy (approval Nr. 16,345/01.07.2025). Written informed consent was obtained from all participants.

### 2.7 Participant Flow

All enrolled participants completed the structured questionnaire and underwent NAAT testing. Serological testing for syphilis, hepatitis B, and hepatitis C was available for 62 of the 70 participants; eight participants could not undergo serology testing due to the absence of health insurance documentation required for laboratory processing within the public hospital system. The analytic denominator for composite outcomes (clinically relevant bacterial STIs) was therefore 62, as this composite required NAAT results and syphilis serology. Complete‐case analysis was used for outcomes requiring serological data; no imputation was performed for missing serology results. All 70 participants completed the questionnaire in full.

## 3 Results

### 3.1 Sample Characteristics

A total of 70 participants were enrolled during the free‐of‐charge testing campaign. The sample was predominantly male (84.3%, *n* = 59) with a mean age of 33.3 ± 11.3 years (range 18–59). The majority identified as MSM (70.0%, *n* = 49), had university education (77.1%), resided in urban areas (88.6%), were single (82.9%), employed (85.7%), and had health insurance (95.7%) (Table 1).

**TABLE 1:** Demographic, behavioral, and clinical characteristics by MSM status (*n* = 70).

Characteristic	Total (*n* = 70)	MSM (*n* = 49)	Non‐MSM (*n* = 21)	*p*
Demographics				
Age, years (mean ± SD)	33.3 ± 11.3	35.2 ± 11.0	29.0 ± 11.0	**0.013** ^**a**^
Male sex, *n* (%)	59 (84.3)	49 (100.0)	10 (47.6)	**< 0.001** ^**a**^
University education, *n* (%)	54 (77.1)	39 (79.6)	15 (71.4)	0.538^a^
Urban residence, *n* (%)	62 (88.6)	44 (89.8)	18 (85.7)	0.689^a^
Single, *n* (%)	58 (82.9)	41 (83.7)	17 (81.0)	0.743^a^
Employed, *n* (%)	60 (85.7)	44 (89.8)	16 (76.2)	0.154^a^
Health insurance, *n* (%)	67 (95.7)	47 (95.9)	20 (95.2)	1.000^a^
Sexual behaviors				
Partners in last 6 months (mean ± SD)	4.8 ± 5.4	6.0 ± 6.1	2.1 ± 1.2	**< 0.001** ^**b**^
> 5 partners in last 6 months, *n* (%)	26 (37.1)	25 (51.0)	1 (4.8)	**< 0.001** ^**c**^
Inconsistent condom use, *n* (%)	38 (54.3)	24 (49.0)	14 (66.7)	0.200^c^
Dating app use, *n* (%)	54 (77.1)	47 (95.9)	7 (33.3)	**< 0.001** ^**c**^
Chemsex, *n* (%)	5 (7.1)	5 (10.2)	0 (0.0)	0.313^c^
Transactional sex, *n* (%)	6 (8.6)	4 (8.2)	2 (9.5)	1.000^c^
Sexual debut < 18 years, *n* (%)	33 (47.1)	21 (42.9)	12 (57.1)	0.306^c^
Testing history and clinical presentation				
Previous STI testing, *n* (%)	56 (80.0)	45 (91.8)	11 (52.4)	**< 0.001** ^**c**^
Previous STI diagnosis, *n* (%)	38 (54.3)	30 (61.2)	8 (38.1)	0.116^c^
Worried about STIs, *n* (%)	42 (60.0)	30 (61.2)	12 (57.1)	0.794^c^
Symptomatic at presentation, *n* (%)	18 (25.7)	10 (20.4)	8 (38.1)	0.143^c^

*Note:* Bold *p* values indicate statistically significant differences between MSM and non‐MSM groups (*p* < 0.05).

Abbreviations: MSM, men who have sex with men; SD, standard deviation; STI, sexually transmitted infection.

^a^ Fisher's exact test.

^b^ Mann–Whitney *U* test.

^c^ Pearson chi‐square test.

Regarding sexual behaviors, the mean number of partners in the past 6 months was 4.8 ± 5.4 (median 3.5, range 1–40), with 37.1% reporting ≥ 5 partners. More than half (54.3%) reported inconsistent condom use, 77.1% used dating apps, 7.1% engaged in chemsex, and 8.6% reported transactional sex. Nearly half (47.1%) had sexual debut before age 18. Testing history showed 80.0% had undergone previous STI testing and 54.3% reported a prior STI diagnosis. At presentation, 25.7% were symptomatic and 60.0% reported worry about contracting an STI after recent sexual encounters.

### 3.2 STI Prevalence

Extensive testing revealed a high overall STI burden (Table 2). Active syphilis was detected in 17.7% (11/62, for which clinical staging identified one primary syphilis, four secondary syphilis, and six latent syphilis cases), HIV in 11.5% (7/61, of which one was de novo and six were people living with HIV), *Chlamydia trachomatis* in 8.6% (6/70), and *Neisseria gonorrhoeae* in 5.7% (4/70). *Mycoplasma genitalium* and *Mycoplasma hominis* were each found in 4.3% (3/70), while *Ureaplasma parvum* was detected in 4.3% (3/70). No cases of *Trichomonas vaginalis* or hepatitis B were detected, and hepatitis C was found in 1.6% (1/62). *Ureaplasma urealyticum* was the most frequently detected organism (21.4%, 15/70) on the multiplex NAAT panel. Of these, 8/15 (53%) were detected alongside a clinically relevant bacterial STI, while 7/15 (47%) represented isolated detection without coinfection. Isolated *Ureaplasma urealyticum* detections were asymptomatic and were not treated. Overall, 35.5% (22/62) of participants had any clinically relevant bacterial STI.

**TABLE 2:** STI prevalence.

Pathogen	*n* tested	*n* positive	Prevalence (%)
*Chlamydia trachomatis*	70	6	8.6
*Neisseria gonorrhoeae*	70	4	5.7
*Mycoplasma genitalium*	70	3	4.3
Syphilis—positive TPHA	62	25	40.3
Active syphilis	62	11	17.7
HIV	61	7	11.5^a^
Hepatitis C	62	1	1.6
Hepatitis B	62	0	0.0
*Trichomonas vaginalis*	70	0	0.0
Composite outcomes			
Any clinically relevant bacterial STI^b^	62	22	35.5
Organisms of uncertain clinical significance^c^			
*Ureaplasma urealyticum*	70	15	21.4
*Mycoplasma hominis*	70	3	4.3
*Ureaplasma parvum*	70	3	4.3

^a^ Includes 1 de novo infection and 6 people living with HIV (PLWH).

^b^ Defined as *Chlamydia trachomatis* , *Neisseria gonorrhoeae* , *Mycoplasma genitalium* , active syphilis.

^c^ Detected as part of multiplex NAAT panel; these bacteria may represent colonization and were not treated in the absence of symptoms and established pathogen coinfection.

Site‐specific analysis revealed important patterns in anatomical distribution (Figure 1). For *Chlamydia trachomatis* , genital infections predominated at 7.2% (5/69), with no pharyngeal cases detected (0/49) and rectal prevalence of 4.8% (1/21). In contrast, *Neisseria gonorrhoeae* demonstrated an inverse distribution with increasing prevalence from genital (1.4%, 1/69) to pharyngeal (4.1%, 2/49) to rectal sites (9.5%, 2/21). Particularly, among 4 participants with *Neisseria gonorrhoeae* , 5 site‐specific infections were identified (one participant had concurrent pharyngeal and rectal infection); 4 of these 5 isolates (80%) occurred at extragenital sites and would have been missed by genital‐only testing.

**FIGURE 1:**
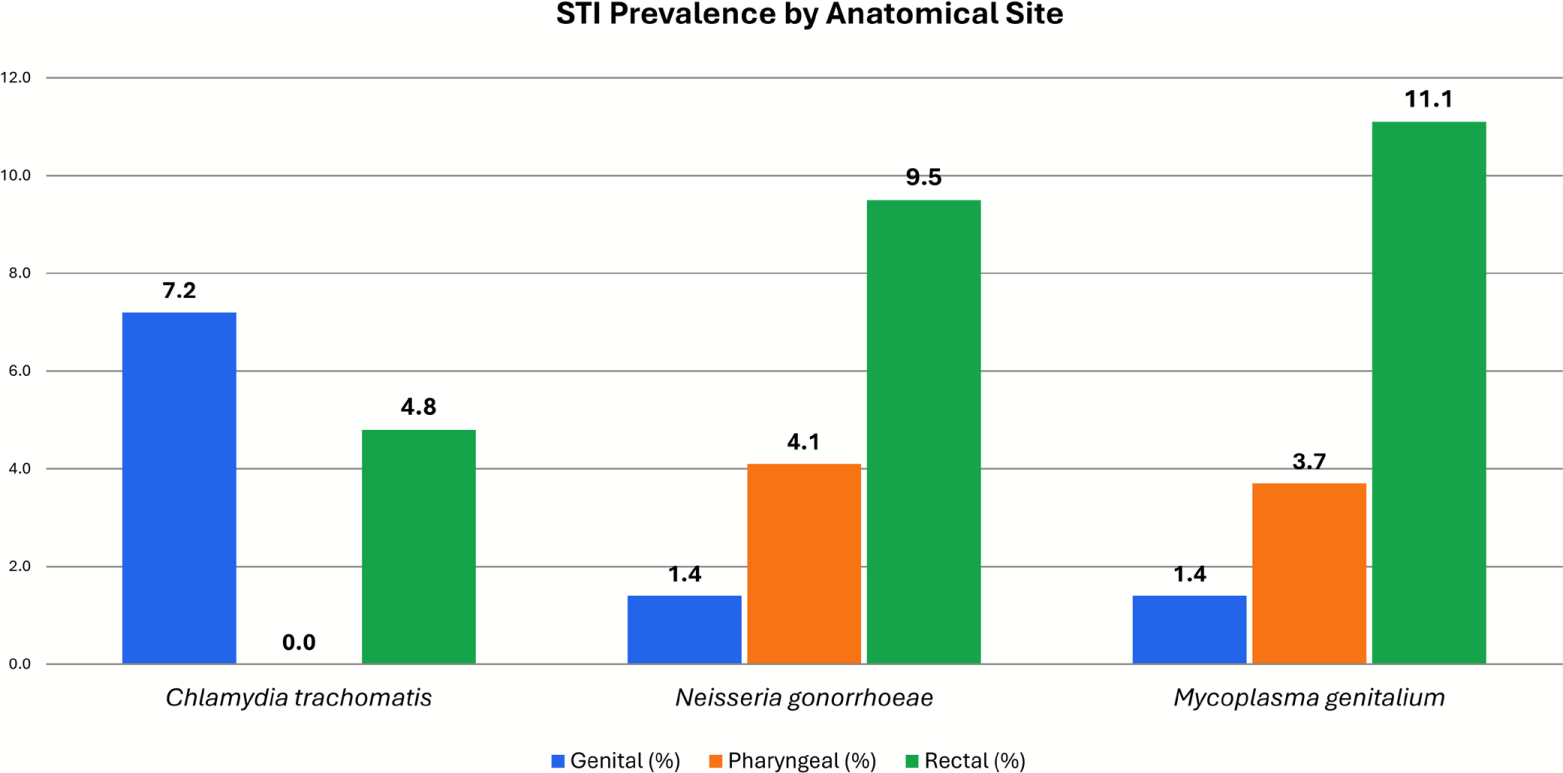
Prevalence of bacterial STI pathogens by anatomical site. Site‐specific prevalence (%) of *Chlamydia trachomatis* , *Neisseria gonorrhoeae* , and *Mycoplasma genitalium* at genital (first‐void urine), pharyngeal, and rectal sites. Sample sizes varied by site based on reported sexual practices.

Among emerging pathogens, *Mycoplasma genitalium* was detected at genital (1.4%, 1/69), pharyngeal (3.7%, 1/27), and rectal sites (11.1%, 1/9), while *Ureaplasma urealyticum* was highly prevalent across all anatomical sites, with genital (13.0%, 9/69), pharyngeal (18.5%, 5/27), and rectal positivity (22.2%, 2/9).

### 3.3 STI Knowledge and Attitudes

Items with highest agreement rates related to condom effectiveness for STI prevention (90%), avoidance of partners with visible lesions (87%), and recognition of syphilis severity (87%). The lowest agreement was observed for recognition of *Chlamydia* and gonorrhea as infections with potentially severe consequences (77% each). For items showing strong consensus toward the favorable response, responses were recoded as binary variables, yielding a total knowledge score ranging from 0 to 17 (mean score: 14.5, representing 85.3% of maximum possible score).

Participants showed low agreement with beliefs such that absence of symptoms indicates absence of infection (mean 2.19 ± 1.81) and that STIs are easy to treat (mean 2.86 ± 1.90), and the item regarding toilet transmission of STIs showed mixed beliefs (mean 4.29 ± 2.32). Participants rated themselves as well‐informed about STIs (mean 5.33 ± 1.48), and high agreement was observed for mandatory comprehensive sex education in schools (mean 6.44 ± 1.23).

MSM showed a trend toward higher overall knowledge (mean rank 38.15 vs. 29.31, *p* = 0.091) and significantly higher knowledge about mother‐to‐child transmission (*p* = 0.001) compared to non‐MSM.

Overall knowledge score did not differ significantly between those with and without STIs (mean rank 26.82 vs. 33.36, *U* = 337.0, *p* = 0.161). Exploratory analysis of individual attitudinal items suggested a pattern warranting further investigation (Figure 2): participants with any clinically relevant bacterial STI scored significantly higher (indicating stronger agreement) on specific items: the belief that absence of symptoms indicates absence of infection (mean rank 37.98 vs. 27.06, *p* = 0.011), the attitude that STIs are not of a personal concern (mean rank 37.75 vs. 27.19, *p* = 0.016), and acceptance of self‐collection as equally effective (mean rank 37.84 vs. 27.14, *p* = 0.019).

**FIGURE 2:**
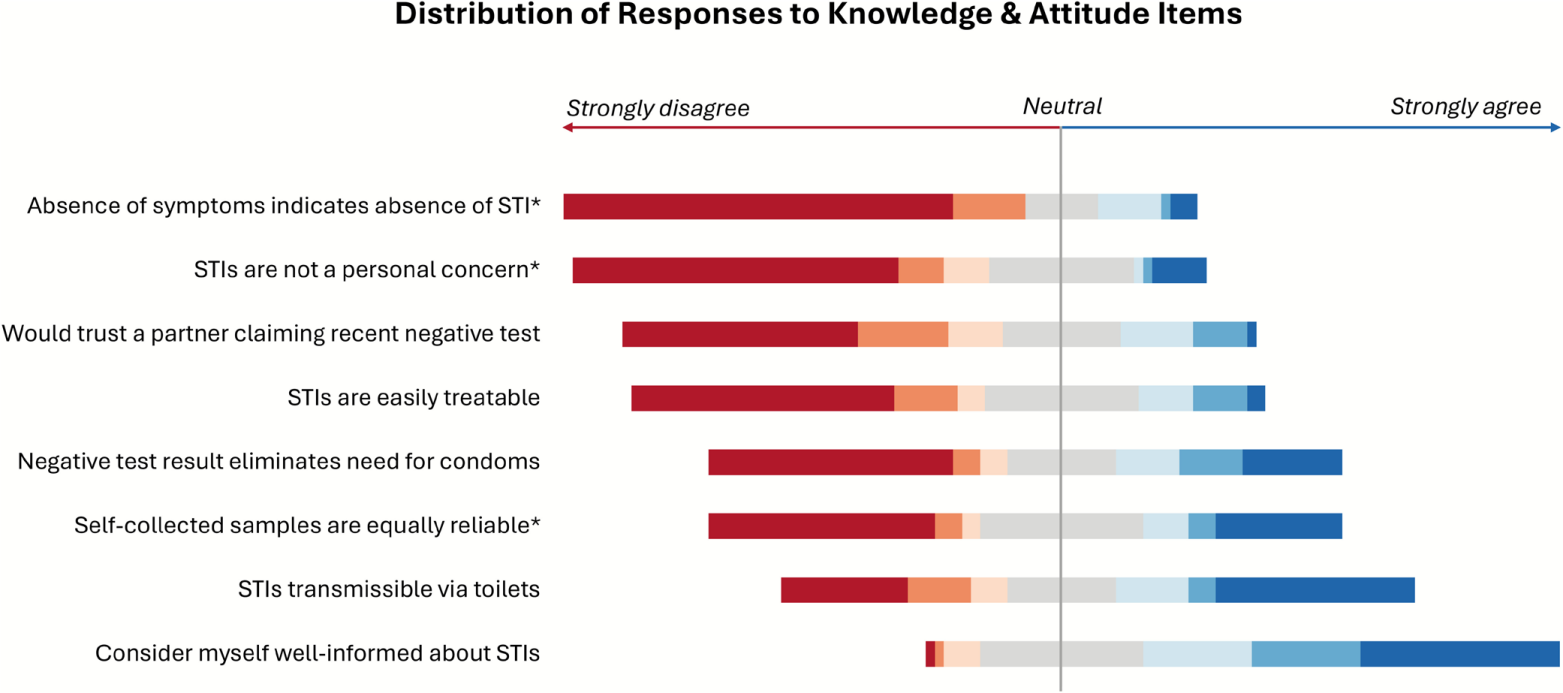
Distribution of responses to knowledge and attitude items (*n* = 70). Bars represent the number of respondents selecting each response option on a 7‐point Likert scale (1 = Strongly disagree to 7 = Strongly agree). Gray bars indicate neutral responses (scored with 4 points on the Likert scale) (4). Items are ordered by mean score. *Items showing significantly higher agreement among participants with any clinically relevant bacterial STI compared to those without (Mann–Whitney *U* test, *p* < 0.05).

### 3.4 STI Determinants

*Chlamydia trachomatis* prevalence was significantly higher in non‐MSM (23.8%, 5/21) compared to MSM (2.0%, 1/49; Fisher's exact test, *p* = 0.008, odds ratio [OR] = 0.067, 95% confidence interval [CI]: 0.007–0.614). The non‐MSM group comprised 11 women and 10 heterosexual men; *Chlamydia trachomatis* was detected in both subgroups: 2/11 (18.2%) in women, 3/10 (30.0%) in heterosexual men. Among the 11 female participants, 4 (36.4%) had a clinically relevant bacterial STI (2 *Chlamydia trachomatis* , 2 active syphilis), comparable to the overall prevalence. Conversely, all *Neisseria gonorrhoeae* cases occurred among MSM, and all occurred in individuals with prior STI history, suggesting repeat infections within high‐risk networks.

Younger age was significantly associated with any bacterial organism (Mann–Whitney *U* = 241.5, *p* = 0.001), and all *Chlamydia trachomatis* cases occurred in participants aged ≤ 34 years (linear‐by‐linear *p* = 0.047).

Partner count was not significantly associated with STI outcomes.

### 3.5 Risk Perception and Testing Preferences

Self‐assessed STI risk showed poor calibration with actual infection status. Only 24.3% of participants rated themselves as high risk, yet 35.5% had a confirmed STI. Self‐perceived risk did not predict actual STI status (χ^2^ = 0.131, *p* = 0.717, Phi = 0.046), indicating widespread underestimation.

Self‐collection methods received neutral‐skeptical acceptance (mean scores 3.0–3.6 on 7‐point scales). No significant differences were observed by MSM status.

A striking difference emerged in preferred screening frequency based on cost: if free‐of‐charge, 62.9% would prefer quarterly testing with only 1.4% preferring symptomatic testing only; if payment was required, only 17.1% would prefer quarterly testing while 35.7% would test only annually (χ^2^ = 25.93, *p* = 0.002). This represents a 45.8 percentage point reduction in preference for optimal screening frequency when cost is introduced. High satisfaction was reported across all service domains: overall satisfaction (77.1%), acceptable waiting time (88.6%), ease of questionnaire completion (85.7%), and respect for confidentiality (88.6%).

## 4 Discussion

The study provides valuable epidemiological data from an underserved region: to our knowledge, this is the first study in Romania to comprehensively examine STI epidemiology within a predominantly MSM cohort while integrating data on sexual behaviors and practices. MSM populations have been largely invisible in Romanian national surveillance data despite their disproportionate STI burden across Europe, making this a significant contribution to sexual health research in this setting. As part of a broader research program investigating gonococcal infection management adapted to individual patient profiles, these findings establish baseline epidemiological parameters essential for subsequent antimicrobial resistance surveillance.

The study was conducted at Colentina Clinical Hospital, a public tertiary care hospital in Bucharest open to all patients regardless of demographic characteristics. The free screening campaign was advertised through social media platforms without targeting specific populations. The predominance of MSM in our sample likely reflects several factors: word‐of‐mouth within MSM networks about the availability of judgment‐free, comprehensive testing; the unique availability of extragenital (pharyngeal and rectal) screening, which is not routinely offered elsewhere in Bucharest; and persistent stigma surrounding STI testing that may motivate MSM to seek specialized services. The lower representation of women likely reflects that in Romania, women typically consult gynecologists for sexual health concerns rather than dermatovenereologists.

Our findings underscore critical systemic issues in Romanian STI surveillance. The high identified prevalence of clinically relevant bacterial STIs highlights a stark discrepancy with national surveillance data, reflecting widespread underreporting. Physicians across specialties frequently fail to report diagnosed STI cases, no mechanisms exist to ensure compliance, and private practitioners often assume reporting responsibilities do not apply to their practice. The absence of a dedicated national STI surveillance network impedes both systematic reporting and epidemiological investigation, rendering national statistics unreliable for public health planning.

The finding of higher *Chlamydia trachomatis* prevalence in non‐MSM compared to MSM warrants cautious interpretation given the heterogeneity of the non‐MSM group, which comprised both women (*n* = 11) and heterosexual men (*n* = 10). *Chlamydia trachomatis* was detected in both subgroups, and genital sampling (first‐void urine) was identical for all participants. In contrast, *Neisseria gonorrhoeae* showed the expected pattern of clustering among MSM with prior STI history.

The importance of extragenital testing deserves particular emphasis. Our finding that the majority of *Neisseria gonorrhoeae* infections were detected at extragenital sites only is consistent with international guidelines recommending pharyngeal and rectal testing for MSM. Standard diagnostic algorithms focusing exclusively on urogenital specimens fail to detect pharyngeal and rectal infections [9], which serve as important reservoirs for ongoing transmission. In Romania, extragenital testing is not routinely available and remains largely inaccessible even to populations at highest risk, compounding the underreporting problem. Our data provide strong support for implementing thorough multisite testing protocols.

While the clinical significance of *Ureaplasma urealyticum* colonization versus infection remains debated [10], our finding that *Ureaplasma urealyticum* was the most commonly detected pathogen, frequent in all anatomical sites, highlights the need for further research to clarify its role in STI syndemics within high‐risk populations. However, nearly half of detections occurred in isolation without clinically relevant coinfections, and these cases were asymptomatic and untreated.

The association between younger age and STI positivity has been consistently observed across settings and likely reflects a combination of biological susceptibility, sexual network characteristics, and less established protective behaviors.

The knowledge–behavior correlations observed in our study have significant implications for STI prevention strategies. While our participants demonstrated proper overall factual knowledge, over one‐third had laboratory‐confirmed bacterial STIs. Those with STIs showed significantly stronger agreement with specific misconceptions, particularly the beliefs that absence of symptoms indicates absence of infections, suggesting that prevention efforts should also focus on specific false beliefs that perpetuate risk behavior. The belief that absence of symptoms indicates absence of infection is particularly concerning given that most STIs are asymptomatic and may lead to delayed testing and ongoing transmission.

The impact of cost on screening preferences has important policy implications. The substantial reduction in preference for quarterly testing when payment is required represents a substantial barrier to achieving recommended screening intervals. The poor calibration between self‐assessed risk and actual STI status is equally alarming: despite over one‐third having confirmed infections, only one‐quarter perceived themselves as high risk. This widespread risk underestimation supports universal screening approaches rather than relying on patient self‐assessment to guide testing decisions.

Romania lacks mandatory comprehensive sexuality education in schools. Current legislation permits only optional “health education” classes requiring parental consent, resulting in low participation rates. Integrating evidence‐based STI education into school curricula should be a public health priority.

Current Romanian national STI guidelines focus predominantly on syndromic management and do not address extragenital screening, asymptomatic testing, or MSM‐specific recommendations. Furthermore, there is no national STI program, and NAAT testing, particularly extragenital testing, is not reimbursed by public health insurance, creating significant financial barriers for uninsured individuals. Updating national guidelines to incorporate routine extragenital screening for at‐risk populations, establishing subsidized or free testing programs, and developing specialized sexual health services could substantially improve STI detection and control. Point‐of‐care testing expansion and the establishment of national reference centers for comprehensive STI screening are the proposed strategies to address these gaps.

### 4.1 Limitations

Our study has several limitations. The sample size of 70 participants limits statistical power for subgroup analyses. As a single‐center study from a specialized clinic in Bucharest, generalizability to other Romanian settings may be limited. The non‐MSM group was heterogeneous, comprising both women and heterosexual men, which limits interpretation of between‐group comparisons. The cross‐sectional design prevents causal inference regarding KAP‐outcome relationships. Selection bias may exist as clinic attendees during a free screening campaign likely represent a higher‐risk population than the general public. Self‐reported behavioral data may be subject to recall and social desirability bias. Multiple comparisons were performed without formal correction, increasing the risk of Type I error.

The comparisons of individual KAP items between participants with and without STIs were exploratory, and no correction for multiple testing was applied. These results should be interpreted as hypothesis‐generating and require confirmation in future studies with pre‐specified hypotheses and larger sample sizes.

Future screening campaigns should enhance outreach to younger populations, who may face additional barriers related to stigma and awareness.

Despite these limitations, the combination of detailed questionnaire data with comprehensive laboratory testing, including often‐overlooked pathogens, offers novel insights into the knowledge–behavior gap and the true burden of STIs in this population.

## 5 Conclusions

This study—the first in Romania to comprehensively examine STI epidemiology in a predominantly MSM cohort, integrated with sexual behavior data—yields the following main findings: (1) STI prevalence was high (35.5% for any clinically relevant bacterial STI), and multisite testing proved essential as 80% of *Neisseria gonorrhoeae* infections would have been missed by genital‐only testing; (2) despite high overall factual knowledge, specific misconceptions persisted across the study population; (3) overall knowledge did not protect against infection—rather, specific false beliefs, particularly that absence of symptoms indicates absence of infection and that STIs are not of personal concern, were significantly associated with positive STI status, while self‐assessed risk poorly predicted actual infection; and (4) cost represents a major barrier to optimal screening frequency, with a substantial reduction in preference for quarterly testing when payment is required, whereas self‐collection methods received only neutral‐skeptical acceptance.

These findings have direct implications for Romanian sexual health policy and service delivery, supporting continued investment in free‐of‐charge screening programs, implementation of comprehensive multisite testing protocols, establishment of a national STI surveillance network to address chronic underreporting, and targeted behavior change interventions addressing specific misconceptions and general knowledge enhancement.

## Funding

This study was conducted within the framework of the URGENT‐STI project (Understanding the Antimicrobial Resistance of Gonorrhea in Europe and Normalizing the Testing for Sexually Transmitted Infections), funded by the European Academy of Dermatology and Venereology (EADV) under the Project Proposal Review Committee grant PPRC‐EADV‐2024‐0024. The free of charge testing campaign was supported by a sponsorship from Reckitt Benckiser (Romania) SRL to the Romanian Association of Dermatology and Venereology, providing funding for laboratory testing and campaign activities.

## Ethics Statement

The study protocol was approved by the Research Ethics Committee of Colentina Clinical Hospital (approval Nr. 2/17.01.2025) and the Scientific Research Ethics Committee of Carol Davila University of Medicine and Pharmacy (approval Nr. 16,345/01.07.2025). Written informed consent was obtained from all participants.

## Conflicts of Interest

George‐Sorin Tiplica received speaker fees from Abbott, Organon, Genesis Biopharma Romania SRL, Leo Pharma Romania, Antibiotice SA, and BMS. Andrei Tanasov: none to declare.

## Supporting information


**Table S1:** Knowledge, attitudes, and practices questionnaire items (*n* = 70).

## Data Availability

The data that support the findings of this study are available from the corresponding author upon reasonable request.
