# Supplementary material for: Sexually Transmitted Infection Prevalence and Determinants Among Sexual Health Clinic Attendees in Bucharest, Romania
Source: Int J Dermatol. 2026 Feb 15;65(12):2554–62. doi: 10.1111/ijd.70344 (PMC13745623; doi:10.1111/ijd.70344)
Supplement: Supplementary file 1 [file IJD-65-2554-s001.docx]

**Supplementary Table 1.** Knowledge, Attitudes, and Practices Questionnaire Items (n=70)

All items were measured using 7-point Likert scales (1 = strongly disagree to 7 = strongly agree). Binary items were dichotomized for calculation of total knowledge score: for correct statements, responses 5-7 coded as 1; for misconceptions, responses 1-3 coded as 1 (correctly rejecting the misconception). Results for binary items show percentage with favorable response (accurate knowledge or protective attitude); results for continuous items show mean ± SD.

| **Item** | **Domain** | **Favorable response** | **Result** |
| --- | --- | --- | --- |
| ***Binary Knowledge Items (included in total knowledge score, range 0-17)*** | | | |
| Gonorrhea, Chlamydia, and syphilis are sexually transmitted infections | Basic knowledge | Agree (5-7) | 100.0% |
| Oral sex is safe because it does not transmit STIs | Transmission misconception | Disagree (1-3) | 82.9% |
| Anal sex is safe because it does not transmit STIs | Transmission misconception | Disagree (1-3) | 85.7% |
| I avoid sexual contact if a potential partner has visible genital lesions | Protective behavior | Agree (5-7) | 87.1% |
| Condoms are an effective method of STI prevention | Prevention knowledge | Agree (5-7) | 90.0% |
| A person who has had gonorrhea, Chlamydia, or syphilis becomes immune and cannot be reinfected | Immunity misconception | Disagree (1-3) | 84.3% |
| I am comfortable discussing sexual health with doctors | Healthcare attitude | Agree (5-7) | 82.9% |
| Untreated gonorrhea can be life-threatening | Disease severity | Agree (5-7) | 77.1% |
| Untreated Chlamydia can be life-threatening | Disease severity | Agree (5-7) | 77.1% |
| Untreated syphilis can be life-threatening | Disease severity | Agree (5-7) | 87.1% |
| Untreated HPV infection can be life-threatening | Disease severity | Agree (5-7) | 82.9% |
| Comprehensive sex education should be mandatory in schools | Prevention attitude | Agree (5-7) | 94.3% |
| Routine STI testing is unnecessary for people outside risk groups | Testing misconception | Disagree (1-3) | 84.3% |
| I would need more sexual partners to be at risk for STIs | Risk misconception | Disagree (1-3) | 85.7% |
| If my test is negative, it means I am not at risk for STIs | Testing misconception | Disagree (1-3) | 78.6% |
| I cannot have an STI because I have no genital symptoms | Symptom misconception | Disagree (1-3) | 85.7% |
| Only people with multiple sexual partners are at risk for STIs | Risk misconception | Disagree (1-3) | 84.3% |
| ***Continuous Items (analyzed individually, 7-point Likert scale)*** | | | |
| If there are no visible lesions or symptoms, the partner most likely does not have an STI | Symptom misconception | Lower score favorable | 2.19 ± 1.81 |
| People can get STIs from using public toilets | Transmission misconception | Lower score favorable | 4.29 ± 2.32 |
| STIs can be transmitted from mother to fetus during pregnancy | Transmission knowledge | Higher score favorable | 5.56 ± 1.77 |
| I usually trust a casual partner who claims to have been recently tested and does not have STIs | Risk behavior attitude | N/A (attitude) | 2.84 ± 1.81 |
| If my partner tells me they have a recent negative STI test, I am likely to have sex without a condom | Risk behavior attitude | N/A (attitude) | 3.54 ± 2.37 |
| Treatment for STIs is accessible | Healthcare perception | N/A (perception) | 4.97 ± 1.98 |
| STIs are easy to treat and do not represent a significant health threat | Severity misconception | Lower score favorable | 2.86 ± 1.90 |
| After these tests, if results are negative, I would show them to future partners and feel entitled to have sex without a condom | Risk behavior attitude | N/A (attitude) | 3.49 ± 2.33 |
| I have discussed my sexual health with my family doctor | Healthcare practice | N/A (practice) | 4.16 ± 2.52 |
| I usually do not ask a new partner about their sexual history | Risk behavior practice | N/A (practice) | 3.17 ± 2.23 |
| I consider myself well-informed about STIs | Self-assessment | N/A (self-assessment) | 5.33 ± 1.48 |
| STIs mainly affect high-risk groups and do not concern me personally | Risk perception | Lower score favorable | 2.54 ± 1.94 |
| ***Self-collection Attitudes (analyzed individually, 7-point Likert scale)*** | | | |
| Self-collection of samples is as effective as collection by a doctor/nurse | Self-collection attitude | N/A (attitude) | 3.57 ± 2.31 |
| If possible, I would prefer to collect samples at home and send them to the laboratory without going to the clinic | Self-collection preference | N/A (preference) | 3.37 ± 2.27 |
| I feel anxious when going to the hospital for sexual health issues | Healthcare anxiety | N/A (attitude) | 3.47 ± 2.28 |
| I am worried about the confidentiality of my STI-related data | Confidentiality concern | N/A (attitude) | 3.03 ± 2.23 |
